# 肺癌合并间质性肺炎药物治疗研究进展

**DOI:** 10.3779/j.issn.1009-3419.2020.102.01

**Published:** 2020-04-20

**Authors:** 言宁 王, 玉皆 周, 立云 苗

**Affiliations:** 210008 南京，南京大学医学院附属鼓楼医院呼吸科 Department of Respiration, Nanjing Drum Tower Hospital, Nanjing 210008, China

**Keywords:** 肺肿瘤, 间质性肺炎, 药物治疗, Lung neoplasms, Interstitial pneumonia, Drug treatment

## Abstract

间质性肺病（interstitial lung disease, ILD）是肺癌发生的危险因素，临床上经常出现肺癌合并间质性肺病（lung cancer associated with ILD, LC-ILD）患者。治疗LC-ILD过程中有导致患者ILD急剧加重甚至死亡的风险，同时合并ILD成为多数肺癌前瞻性临床试验的排除标准，因此当肺癌合并ILD时常常成为肺癌的治疗难点。由于LC-ILD患者在临床中占有一定的比例，因此有必要探寻其最佳的治疗方案。在此我们综述了现有的临床研究结果以供参考。

## Abstract (en)

Interstitial lung disease (ILD) is a risk factor for lung cancer. Patients with lung cancer associated with ILD (LC-ILD) often appear clinically. During the treatment of LC-ILD, there is a risk of causing acute exacerbation or even death in the treatment of lung cancer. At the same time, combining ILD has become the exclusion criteria for prospective clinical trials of most lung cancers. Therefore, when lung cancer is combined with ILD, it often becomes a difficult point for the treatment of lung cancer. Because LC-ILD patients have a certain proportion in the clinic, it is necessary to explore the best treatment options. Here we review the results of existing clinical studies for reference.

## 1 前言

间质性肺病（interstitial lung disease, ILD）是一种呼吸功能进行性恶化的疾病^[1]^。ILD是肺癌发生的独立危险因素，在被诊断为肺癌的患者中有5.8%-15.2%的患者合并有ILD^[2, 3]^。流行病学研究^[4]^表明多达22%的ILD患者最终发展为肺癌，其风险约是普通人群的5倍。ILD与肺癌之间有着共同的致病机制，在成纤维细胞形成过程中，基因（*p53*、*SFTPA1*、*SFTPA2*）突变，可溶性介质[转化生长因子（transforming growth factor, TGF）、一氧化氮（nitric oxide, NO）、活性氧（reactive oxygen species, ROS）]释放，细胞凋亡失调，使上皮细胞发生癌性转化^[5]^。虽然ILD与肺癌之间存在广泛的流行病学和机制联系，但是对这类特殊患者的治疗却缺乏更为广泛的深入探讨。虽然ILD的临床进展比较缓慢，但是其中一些患者会出现ILD的急性加重（acute exacerbation, AE），通常表现为突然进行性和严重的呼吸衰竭，伴有新的肺部混浊和弥漫性肺泡损伤的病理病变^[6]^。由于目前没有明确的AE-ILD治疗方案，因此AE-ILD患者的预后很差。在肺癌合并间质性肺病（lung cancer associated with ILD, LC-ILD）患者中，各种抗癌治疗都可能诱导AE-ILD的发生^[7]^。虽然合并ILD的早期肺癌患者可以通过手术进行治疗，但术后有AE-ILD的风险^[8]^。鉴于放疗在LC-ILD患者中有着较高的AE风险，这类患者应避免使用局部放疗。由于大多数肺癌的药物临床试验均排除了ILD患者，因此目前仍没有LC-ILD的标准药物治疗方案。在此，我们检索有关LC-ILD药物治疗的文献，并对现有的治疗方案做一综述以供参考。

## 2 细胞毒性药物

来自Chen等^[9]^的一项*meta*分析纳入了7项研究总共251例患者，研究显示接受化疗的非小细胞肺癌（non-small cell lung cancer, NSCLC）合并ILD患者的客观缓解率（objective response rate, ORR）为41.3%，无进展生存期（progression-free survival, PFS）为4.4个月，总生存期（overall survival, OS）为8.5个月，AE-ILD的发生率为8.47%。该研究表明化疗是NSCLC-ILD患者的有效治疗方法，但化疗可能与较高的AE-ILD发生率有关。最近在Kawahara等^[10]^的研究中，进行化疗的LC-ILD患者相较于仅进行最佳支持治疗（best supportive care, BSC）的LC-ILD患者有更长的OS（14.3个月*vs* 7.2个月），并且BSC组患者均可接受化疗，表明化疗在这类患者中是有效的。由于AE-ILD是影响LC-ILD患者生存的首要因素，因此在对这类患者进行化疗之前，评估AE-ILD的发生风险非常重要。Kenmotsu等^[11]^回顾性分析了109例LC-ILD患者，发现普通间质性肺炎患者（usual interstitial pneumonia, UIP）比非UIP患者的化疗相关AE-ILD发生率更高（30% *vs* 8%, *P*=0.005）。具有UIP模式的患者对类固醇药物和免疫抑制剂的敏感性都更低，并且有着更高的AE-ILD死亡率。因此，针对UIP模式的肺癌患者，化疗时需要密切监测以防AE-ILD的发生。

### 2.1 NSCLC-ILD患者的一线化疗

目前仅有5项前瞻性研究调查了晚期NSCLC-ILD患者一线使用化疗药物使用的有效性及安全性，在这5项研究中，NSCLC-ILD患者一线化疗的ORR、PFS和安全性均与不合并ILD的患者无显著差异。Minegishi等^[12]^的研究总共纳入18例NSCLC-ILD患者，采用卡铂加紫杉醇的治疗方案。研究显示ORR为61.1%，PFS为5.3个月，OS为10.6个月，其中1例UIP型患者在化疗4个周期后出现AE-ILD，并且在8周后死亡。而Sekine等^[13]^的研究纳入21例接受卡铂加S-1治疗的NSCLC-ILD患者，其中有2例患者出现了AE-ILD，但是这2例患者的AE-ILD均控制良好。另外Hanibuchi的研究^[14]^也表明一线卡铂联合S-1治疗NSCLC-ILD患者是安全有效的。

一项Ⅲ期临床试验^[15]^阐明，在晚期NSCLC患者中卡铂联合白蛋白紫杉醇比联合常规紫杉醇有更好的ORR（33% *vs* 25%, *P*=0.005）和安全性。截至目前，有两项前瞻性试验探究了白蛋白紫杉醇在NSCLC-ILD患者中的有效性及安全性。Kenmotsu等^[16]^的研究共纳入92例NSCLC-ILD患者，OS达到15.4个月，有4例（4.3%）患者出现了ILD加重，其中1例患者死亡。Asahina等^[17]^的研究纳入了36例NSCLC-ILD患者，OS为15.4个月，AE-ILD发生率为5.6%。这两项研究表明，卡铂联合白蛋白紫杉醇方案相比其他化疗方案有着更低的AE-ILD发生率和更好的OS，是更安全有效的化疗方案。同时，多项回顾性研究^[18-21]^显示卡铂联合白蛋白紫杉醇治疗晚期NSCLC-ILD的AE-ILD发生率为0.0%-8.3%，近似于不合并肺癌的ILD患者在自然进程下的AE发生率（8.5%），表明卡铂联合白蛋白紫杉醇在NSCLC-ILD患者中有较好的安全性。因此，白蛋白紫杉醇相较常规紫杉醇及其他化疗方案可能是NSCLC-ILD患者一线化疗的更好选择。

培美曲塞和吉西他滨分别是驱动基因阴性的晚期非鳞NSCLC和晚期肺鳞癌一线化疗的首选化疗药物。在Fujita等^[22]^的研究中，共有24例NSCLC-ILD患者接受培美曲塞联合铂类一线化疗，ORR为33.3%，PFS为4.7个月，OS为9.5个月，有3例（12.5%）患者一线化疗期间出现AE-ILD，并且其中1例患者死亡。Choi等^[23]^的研究纳入13例使用铂类联合培美曲塞和39例使用铂类联合吉西他滨的NSCLC-ILD患者。结果显示培美曲塞组的AE-ILD发生率达15.4%，并且患者均死亡，而吉西他滨组的AE-ILD发生率为2.6%。可见培美曲塞联合铂类一线治疗NSCLC-ILD患者比其他化疗方案的AE-ILD发生率更高，不推荐此方案应用于NSCLC-ILD患者。

Kenmotsu等^[24]^研究NSCLC-ILD患者使用含铂双药方案的预后，发现104例患者的总生存期为9.9个月，低于不合并ILD的NSCLC患者的11个月-15个月，表明NSCLC-ILD患者有着更差的预后，并且发现肿瘤的临床分期是NSCLC-ILD患者的独立预后因素。值得注意的是，在以上的所有研究中，NSCLC-ILD患者一线化疗的ORR和PFS都与无ILD的NSCLC患者相似，但是OS较低，这可能与二线化疗中患者的肺功能恶化和较高的AE发生率有关^[25-27]^（表 1）。

**1:** LC-ILD患者一线化疗的前瞻性研究 Prospective study of first-line chemotherapy in patients with LC-ILD

Author	Histology	*n*	Regimen	ORR (%)	mPFS (mon)	mOS (mon)	AE[*n*(%)]
Minegishi^[12]^	NSCLC	18	CBP+PTX	61.1	5.3	10.6	1 (5.6)
Sekine^[13]^	NSCLC	21	CBP+S-1	33.3	4.2	9.7	2 (9.5)
Hanibuchi^[14]^	NSCLC	33	CBP+S-1	33.3	4.8	12.8	2 (6.1)
Kenmotsu^[16]^	NSCLC	92	CBP+nab-PTX	51.0	6.2	15.4	4 (4.3)
Asahina^[17]^	NSCLC	36	CBP+nab-PTX	55.6	5.3	15.4	2 (5.6)
Watanabe^[30]^	SCLC	17	CBP+VP-16	88.2	5.5	8.7	1 (5.9)
ORR: objective response rate; mPFS: median progression-free survival; mOS: median overall survival; AE: acute exacerbation of ILD; CBP: carboplatin; PTX: paclitaxel; VP-16: etoposide; LC-ILD: lung cancer associated with interstitial lung disease; nab: albuminbound.							

### 2.2 SCLC-ILD患者的一线化疗

Togashi等的研究^[28]^发现SCLC-ILD患者一线化疗的ORR和PFS与不合并ILD的SCLC患者相比没有显著差异，表明SCLC-ILD患者可以从化疗中受益，但是SCLC-ILD患者的OS明显更差（10.7个月*vs* 17.8个月，*P*=0.001）多变量分析后发现预先存在ILD是患者OS较差的独立危险因素。另外两项回顾性研究^[29, 30]^均表明SCLC-ILD患者可从一线铂类联合依托泊苷化疗中获益。一项前瞻性研究^[31]^表明卡铂联合依托泊苷治疗SCLC-ILD患者是安全有效的，但需要更多大规模的临床试验来确定AE-ILD发生的危险因素。

### 2.3 LC-ILD患者的二线化疗

在一项纳入278例LC-ILD患者（NSCLC, *n*=204; SCLC, *n*=74）的研究中^[32]^，有72例NSCLC-ILD患者进行二线多西他赛单药治疗，其中11例（15.3%）发生AE-ILD。一些回顾性研究^[33^, 34^]^发现使用多西他赛和培美曲塞进行二线单药治疗，AE-ILD的发生率分别为12.0%和14.3%，这些研究表明多西他赛与培美曲塞单药化疗具有较差的疗效和较高的肺毒性，不推荐作为此类患者的二线治疗方法。

Nokihara等发现^[35]^紫杉醇和S-1在NSCLC-ILD患者的一线化疗中，AE-ILD发生率相似。而在二线化疗中，紫杉醇的AE-ILD发生率从一线的8.3%上升至15.2%，在使用S-1的患者中却没有发生AE-ILD。并且卡铂联合S-1一线治疗NSCLC-ILD的有效性及安全性已在前瞻性研究中得到验证，考虑到一线及二线化疗的AE-ILD发生率，卡铂联合S-1在NSCLC-ILD人群中，可能是优于其他主流化疗方案的治疗选择。

Suzuki等^[36]^的研究表明接受拓扑替康二线化疗的SCLC-ILD患者比仅接受一线化疗的患者有着更长的总生存期，拓扑替康二线化疗在这类患者中是可行的。而在Enomoto等^[37]^的研究中拓扑替康二线化疗的安全性较差，AE-ILD的发生率明显高于Suzuki的研究（21.7% *vs* 8.3%）。Saijo等^[38]^研究了紫杉醇二线治疗SCLC-ILD的有效性和安全性，结果表明紫杉醇对这类患者具有较好的抗肿瘤活性，但是由于AE发生率较高（29.4%），患者的生存获益受限。

## 3 酪氨酸激酶抑制药

近年来，有关NSCLC驱动基因的研究取得了一定进展。60%-70%的NSCLC患者可检测出驱动基因突变，促成了酪氨酸激酶抑制剂（tyrosine kinase inhibitors, TKI）的发展^[39]^。虽然在有驱动基因突变的患者中，靶向治疗比传统化疗更有效，但有系统评价^[40, 41]^表明表皮生长因子受体（epidermal growth factor receptor, EGFR）抑制剂吉非替尼和厄洛替尼暴露与ILD的风险显著增加有关（RR=1.53），其中有1.5%的患者发生吉非替尼相关的ILD，0.9%的患者发生厄洛替尼相关的ILD。一项临床研究^[42]^评估了*EGFR*突变肺癌患者接受TKI治疗的安全性，结果显示亚洲人群比非亚洲人群更易发生药物治疗相关ILD（2.5% *vs* 0.9%），而日本人群对比非日本人群有更高的靶向药物治疗相关ILD发生率（3.8% *vs* 0.3%）。同时，日本的大规模对照试验^[43]^发现先前存在ILD与靶向药物治疗诱导的肺炎具有高度的相关性（OR=2.89）。因此来自亚洲特别是日本的NSCLC-ILD患者应谨慎使用EGFR-TKI，以防止AE-ILD的发生。

Yoneda等^[44]^发现在亚洲NSCLC患者中间变性淋巴瘤激酶（anaplastic lymphoma kinase, ALK）抑制剂诱导的ILD发生率为1.2%，而在日本人群中ALK抑制剂诱导的ILD发生率达到3.7%。一项来自日本的真实世界研究^[45]^总共纳入2, 028例使用克唑替尼的NSCLC患者，其中117例（5.77%）患者发生了克唑替尼相关的ILD，并且年龄 > 55岁、体力评分2分-4分、吸烟史和先前存在ILD被认定为克唑替尼诱导ILD的危险因素，而先前存在ILD是最关键的危险因素（HR=12.6）。

## 4 免疫检测点抑制剂

Nivolumab是一种人免疫检验点抑制药抗体，可抑制PD-1受体，提高抗肿瘤免疫力。在CheckMate 057研究^[46]^中，PD-1单抗Nivolumab较标准二线化学治疗药物多西他赛可显著延长晚期非鳞状NSCLC患者的总生存期。但是由于担心PD-1单抗会导致LC-ILD患者病情恶化，临床医生一般避免给予这类患者免疫治疗。最近，一些回顾性研究发现^[47, 48]^先前存在ILD会显著增加免疫治疗相关肺炎发生的风险，同时会更早发生肺炎（1.3个月*vs* 2.3个月），但是这部分患者的生存结局较好，这可能和较高的激素治疗应答率有关。另外多项研究^[49-51]^表明免疫治疗相关不良反应（包括肺炎）和肺癌治疗的临床获益有关。而PD-1相关性肺炎对比吉非替尼相关性肺炎有着更高的激素治疗应答率和更低的死亡率^[52, 53]^。一些案例^[54-56]^报道，使用PD-1单抗治疗的LC-ILD患者没有出现ILD恶化，并且部分患者获得了持续的免疫应答。在一项评估6例NSCLC-ILD患者使用Nivolumab的安全性研究^[57]^中，没有患者发生AE-ILD，并且有3例患者出现部分缓解。因此我们认为虽然在LC-ILD患者中免疫相关肺炎的发生风险更高，但是免疫相关肺炎是可以控制的，若在治疗早期做好密切监测，免疫治疗有望成为这部分患者的治疗选择。

## 5 抗血管生成药物

最近，血管内皮生长因子（vascular endothelial growth factor, VEGF）被认为在AE-ILD的发病过程中扮演着重要角色。新血管生成是组织损伤后愈合的基本过程。在实验室肺纤维化中，可观察到血管生成和VEGF活性增加^[58]^。在动物实验中，通过抑制VEGF可减少博来霉素诱导的肺损伤和纤维化^[59]^。但其他研究^[60]^发现，成纤维细胞灶内几乎没有毛细血管，表明纤维化不需要新生血管生成。因此，目前尚不清楚VEGF是否促进肺纤维化。

贝伐珠单抗是全球第一个上市的抗肿瘤VEGF单克隆抗体药物，并且是目前唯一批准用于晚期或复发性NSCLC一线治疗的抗血管生成药物^[61]^。两项回顾性研究^[62, 63]^表明卡铂和紫杉醇联合贝伐珠单抗是安全有效的，并且没有增加NSCLC-ILD患者AE-ILD的发生率。最近一项研究^[64]^表明一线化疗联合贝伐珠单抗可以降低NSCLC-ILD患者化疗相关AE-ILD的风险（0% *vs* 22.6%, *P*=0.037），并且贝伐珠单抗组对比非贝伐珠单抗组有着更长的PFS（8.0个月*vs* 4.3个月，*P*=0.026）（表 2）。

**2:** LC-ILD患者一线化疗的回顾性研究 Retrospective study of first-line chemotherapy in patients with LC-ILD

Author	Histology	*n*	Regimen	ORR (%)	mPFS (mon)	mOS (mon)	AE[*n*(%)]
Niwa^[18]^	NSCLC	9	CBP+nab-PTX	55.6	5.7	11.3	0 (0.0)
Yasuda^[19]^	NSCLC	12	CBP+nab-PTX	67	5.1	14.9	1 (8.3)
Araya^[20]^	NSCLC	9	CBP+nab-PTX	77.8	5.8	8.0	0 (0.0)
Fujita^[21]^	NSCLC	8	CBP+nab-PTX	50	5.6	8.1	0 (0.0)
Fujita^[22]^	NSCLC	24	PT+PEM	33.3	4.7	9.5	3(12.5)
Choi^[23]^	NSCLC	13	PT+PEM	38.5	6.1	7.9	2(15.4)
		39	PT+GEM	43.6	5.1	7.9	1(2.6)
Kenmotsu^[24]^	NSCLC	104	PT+any	38	4.8	9.9	9 (8.7)
		63	(CBP+PTX)	-	4.4	8.2	5 (7.9)
Okuda^[25]^	NSCLC	19	PT+VNR	42.1	4.4	7.4	3 (15.8)
Watanabe^[26]^	NSCLC	67	PT+VNR	34.3	3.7	7.4	7 (10.4)
Shukuya^[27]^	NSCLC	15	CBP+PTX	33	2.5	7.0	4 (26.7)
Yoshida^[29]^	SCLC	52	PT+VP-16	69	4.5	9.4	1 (1.9)
Watanabe^[30]^	SCLC	11	PT+VP-16	63.6	4.7	7.0	3 (27.3)
Shimizu^[62]^	NSCLC	11	CBP+PTX	27	4.4	9.7	0 (0.0)
		10	CBP+PTX+Bev	40	5.3	16.1	1 (10.0)
Enomoto^[63]^	NSCLC	25	CBP+PTX+Bev	72	7.2	8.5	3 (12.0)
Hamada^[64]^	NSCLC	17	CBP+PEM+Bev	-	8.0	-	0 (0.0)
		31	CBP+PEM	-	4.3	11.2	7 (22.6)
Bev: bevacizumab; PEM: pemetrexed; VNR：vinorelbine.							

尼达尼布是一种小分子三联血管激酶抑制剂，靶向血管内皮生长因子受体，血小板衍生生长因子受体以及成纤维细胞生长因子受体。Ⅱ期/Ⅲ期临床试验^[65, 66]^已经证明了该抑制剂治疗特发性肺纤维化（idiopathic pulmonary fibrosis, IPF）患者的疗效和安全性。LUME-Lung 1研究^[67]^评估了多西他赛联合尼达尼布作为NSCLC二线治疗的疗效和安全性，该研究结果表明，尼达尼布联合多西他赛是晚期NSCLC患者的有效二线治疗方案，尤其是对于腺癌患者。而尼达尼布联合化疗在LC-ILD患者中的疗效及安全性尚不清楚，目前有一项Ⅱ期随机对照J-SONIC研究^[68]^正在进行中，该研究旨在探究尼达尼布联合卡铂和白蛋白紫杉醇治疗LC-IPF，是否可以延长患者发生AE-IPF的时间间隔，未来这项研究的结果可能为LC-ILD患者提供新的治疗选择。Yamakawa等^[69]^报道了1例SCLC-ILD患者在先后接受帕博利珠单抗和阿特利珠单抗治疗后都发生了免疫相关ILD，患者在加用尼达尼布后再次挑战使用阿特利珠单抗治疗，治疗3个周期后患者病情稳定，没有发生AE-ILD。

## 6 其他药物

吡非尼酮是一种口服小分子化合物，具有抗炎和抗氧化的多效性。三项Ⅲ期临床试验^[70, 71]^的结果显示，吡非尼酮组与安慰剂组相比，延缓了IPF患者的疾病进展，延长了患者的总生存期。2014年，吡非尼酮在美国获批用于治疗IPF。在临床前模型中^[72]^，吡非尼酮联合顺铂导致癌症相关成纤维细胞和NSCLC细胞死亡增加。最近，一项回顾性研究^[73]^表明IPF患者使用吡非尼酮可以降低肺癌的发生率（2.4% *vs* 22.0%, *P* < 0.000, 1）。来自日本的单臂Ⅱ期临床研究^[74]^表明，围手术期使用吡非尼酮有望降低肺癌术后AE-IPF的发生率。这些研究结果显示了吡非尼酮同时在治疗肺癌和肺纤维化方面的潜力，可为LC-ILD患者的治疗提供新的思路。

## 7 小结

虽然缺少大规模的前瞻性临床研究，但是基于目前的临床数据，卡铂联合白蛋白紫杉醇治疗NSCLC-ILD相较于其他化疗方案有着更好的疗效和更低的AE-ILD发生率，推荐用于这类患者的一线治疗。二线化疗的AE-ILD风险较高，预后较差，并且可能是患者OS不理想的原因。由于TKI相关ILD的发生率较高，目前缺乏LC-ILD患者使用TKI的研究。免疫抑制剂相关的ILD是可以控制的，可以用于LC-ILD患者的治疗。多项研究显示抗血管生成药物联合化疗可以降低AE-ILD发生率，在LC-ILD患者中表现出巨大的应用前景。未来需要进行更多的临床试验，以探究抗血管生成药物联合免疫抑制剂或者TKI治疗LC-ILD的安全性及有效性，为LC-ILD患者制定合理有效的治疗方案，获得更好的预后。
